# Exposure-based treatments for childhood abuse-related post-traumatic stress disorder in adults: a health-economic evaluation

**DOI:** 10.1080/20008066.2023.2171752

**Published:** 2023-02-14

**Authors:** Marie-Louise J. Kullberg, Maartje Schoorl, Danielle A. C. Oprel, Chris M. Hoeboer, Filip Smit, Willem van der Does, Rianne A. de Kleine, Agnes van Minnen, Wilbert van den Hout

**Affiliations:** a Department of Clinical Psychology, Leiden University, Leiden, The Netherlands; b PsyQ, Parnassiagroep, The Hague, The Netherlands; c Department of Psychiatry, Amsterdam University Medical Centers Location AMC, Amsterdam Public Health, Amsterdam, The Netherlands; d Centre of Health Economic Evaluation and Department of Mental Health and Prevention, Trimbos Institute (Netherlands Institute of Mental Health and Addiction), Utrecht, The Netherlands; e Department of Clinical Psychology and Department of Epidemiology and Biostatistics, University Medical Centers Amsterdam, Location VUmc, Amsterdam, The Netherlands; f PSYTREC, Bilthoven, The Netherlands; g Behavioural Science Institute, Radboud University, Nijmegen, The Netherlands; h Department of Biomedical Data Sciences, Leiden University Medical Center, Leiden, The Netherlands

**Keywords:** PTSD, cost-effectiveness, prolonged exposure, childhood abuse, net-benefit analysis, TEPT, costo-efectividad, exposición prolongada, abuso infantil, PTSD, 成本效益, 延长暴露, 童年期虐待

## Abstract

**Background:** Prolonged exposure (PE) is an effective treatment for post-traumatic stress disorder (PTSD).

**Objective:** This study aimed to analyse the cost-effectiveness of three exposure-based treatments in patients with childhood abuse-related PTSD.

**Method:** A net–benefit analysis was conducted alongside a pragmatic randomized controlled trial with participants (*N *= 149) randomized to three conditions: PE (*n = *48), intensified PE (i-PE, *n *= 51), and phase-based PE [Skills Training in Affective and Interpersonal Regulation (STAIR) + PE, *n *= 50]. Assessments took place at baseline (T0), post-treatment (T3), 6 month follow-up (T4), and 12 month follow-up (T5). Costs stemming from healthcare utilization and productivity losses were estimated using the Trimbos/iMTA questionnaire for Costs associated with Psychiatric Illness. Quality-adjusted life-years (QALYs) were based on the 5-level EuroQoL 5 Dimensions (EQ-5D-5L) using the Dutch tariff. Missing values of costs and utilities were multiply imputed. To compare i-PE to PE and STAIR + PE to PE, pair-wise unequal-variance *t*-tests were conducted. Net–benefit analysis was used to relate costs to QALYs and to draw acceptability curves.

**Results:** Intervention costs did not differ across the three treatment conditions. Total medical costs, productivity losses, total societal costs, and EQ-5D-5L-based QALYs did not differ between treatment conditions either (all *p* > .10). At the relevant €50,000/QALY threshold, the probability of one treatment being more cost-effective than another was 32%, 28%, and 40% for PE, i-PE, and STAIR-PE, respectively.

**Conclusion:** Three equally effective treatments were compared and no differences in cost-effectiveness between treatments were found. Therefore, we advocate the implementation and adoption of any of the treatments and endorse shared decision making.

## HIGHLIGHTS

- This is the first study to compare cost-effectiveness of three exposure-based treatments in patients with CA-PTSD alongside a randomized controlled clinical trial (*N* = 149).
- The three exposure-based treatments did not differ in terms of outcomes and costs.
- Findings underline that any of these treatments can be implemented, and we endorse shared decision making to meet patient treatment preference.

## Abstract (es)

**Objetivo:** Este estudio tuvo como objetivo analizar el costo-efectividad de tres tratamientos basados en la exposición en pacientes con trastorno de estrés postraumático relacionado con el abuso infantil (TEPT-CA).

**Método:** Se llevó a cabo un análisis de beneficio neto junto con un ensayo controlado aleatorio pragmático (ECA) con participantes (*N* = 149) asignados al azar a tres condiciones; exposición prolongada (PE, *n* = 48), exposición prolongada intensificada (i-PE, *n* = 51) y exposición prolongada basada en fases (STAIR + PE, *n* = 50). Las evaluaciones se realizaron al inicio (T0), después del tratamiento (T3), a los seis meses de seguimiento (T4) ya los doce meses de seguimiento (T5). Los costos derivados de la utilización de la atención médica y las pérdidas de productividad se estimaron utilizando el Cuestionario de costos para trastornos psiquiátricos (TiC-P) de Trimbos e iMTA. Los años de vida ajustados por calidad (AVAC) se basaron en el EuroQoL de 5 niveles (EQ-5D-5L) utilizando los costos de vida holandeses. Los valores faltantes de costos y utilidades se imputaron de forma múltiple. Para comparar i-PE con PE y STAIR + PE con PE, se realizaron pruebas t de varianza desigual por pares. Se utilizó el análisis de beneficio neto para relacionar los costos con los AVAC y para graficar las curvas de aceptabilidad.

**Resultados:** Los costos de la intervención no difirieron entre las tres condiciones de tratamiento. Los costes médicos totales, las pérdidas de productividad, los costes sociales totales y los AVAC basados en EQ-5D-5L tampoco difirieron entre las condiciones de tratamiento (todos los valores de *p* > 0,10). En el umbral correspondiente de 50,000 euros por QALY, la probabilidad de que un tratamiento fuera más rentable que otro era del 32%, 28% y 40% para PE, i-PE y STAIR-PE, respectivamente.

**Conclusión:** Se compararon tres tratamientos igualmente efectivos y no se encontraron diferencias en el costo-efectividad entre tratamientos. Por lo tanto, defendemos la implementación y adopción de cualquiera de estos tratamientos y respaldamos la toma de decisiones compartida.

## Abstract (zh)

**目的：** 本研究旨在分析三种基于暴露的治疗对童年期虐待相关创伤后应激障碍 (CA-PTSD) 患者的成本效益。

**设计：**净收益分析与实用随机对照试验 (RCT) 一起进行，参与者 (*N* = 149) 随机分配到三种情况； 延长暴露（PE，*n* = 48）、强化延长暴露（i-PE，*n* = 51）和阶段性延长暴露（STAIR + PE，*n* = 50）。 在基线 (T0)、治疗后 (T3)、六个月随访 (T4) 和十二个月随访 (T5) 时进行评估。

**测量：**使用 Trimbos 和 iMTA 精神障碍成本问卷 (TiC-P) 估算了因医保利用和生产力损失而产生的成本。 质量调整生命年 (QALY) 基于使用荷兰关税的 5 级 EuroQoL (EQ-5D-5L)。

**分析：**成本和公用事业的缺失值被多重插补。 为了比较 i-PE 与 PE 和 STAIR + PE 与 PE，进行了成对不等方差 t 检验。 使用净效益分析将成本与 QALY 相关联并绘制可接受性曲线。

**结果：**干预成本在三种治疗条件下没有差异。 总医疗成本、生产力损失、总社会成本和基于 EQ-5D-5L 的 QALY 在治疗条件之间也没有差异（所有 p 值 > 0.10）。 在相对 50.000 欧元/QALY 阈值下，PE、i-PE 和 STAIR-PE 比其他治疗更具成本效益的概率分别为 32%、28% 和 40%。

**结论：**比较了三种同样有效的治疗方法，发现治疗方法之间的成本效益没有差异。 因此，我们提倡实施和采用任何治疗方法，并支持共同决策。

## 1. Introduction

Prolonged exposure (PE) is an effective treatment for post-traumatic stress disorder (PTSD) (Martin et al., 2021; Mavranezouli et al., 2020; Watkins, Sprang & Rothbaum, 2018), and is listed as one of the preferred interventions in treatment guidelines (Card, 2017; Hamblen et al., 2019). We recently examined the effects of three variants of PE in patients with childhood abuse-related post-traumatic stress disorder (CA-PTSD). Compared with patients with PTSD related to trauma in adulthood, patients with CA-PTSD improve less on PTSD symptoms, emotion regulation, and interpersonal functioning (Karatzias et al., 2019). CA-PTSD is also associated with higher dropout rates during trauma-focused treatment (Ehring et al., 2014). As such, we compared standard PE to two adaptations of PE, intensified prolonged exposure (i-PE) and phase-based treatment, in a randomized controlled trial (RCT) to examine whether these adaptations improve treatment outcomes (IMPACT study) (Oprel et al., 2018). i-PE has previously been found to lead to fast symptom reduction and low dropout rates in PTSD (Foa et al., 2018; Hendriks et al., 2018), but has not yet been tested in a controlled trial in patients with CA-PTSD. Phase-based treatment means that PE is preceded by Skills Training in Affective and Interpersonal Regulation (STAIR). STAIR is based on the notion that emotion regulation problems and interpersonal difficulties interfere with processing of the traumatic memories and hinder the treatment effects of PE (Cloitre et al., 2002). STAIR + PE has been found to be effective in CA-PTSD (Cloitre et al., 2010).

PTSD guidelines (APA, 2017) and Guidelines for the Management of Conditions Specifically Related to Stress (WHO, 2013) highlight the need for cost-effectiveness analyses to complement findings from RCTs on clinical treatment outcomes. Information on cost-effectiveness may support guideline panels, financiers, policy makers, and clinicians in decision making. Hence, we conducted a cost–utility analysis alongside our RCT, to determine which of the three conditions is preferred in terms of 1 year healthcare and societal costs and quality-adjusted life-years (QALYs). A recent systematic review (von der Warth et al., 2020) included 18 economic evaluations of therapeutic interventions for PTSD. The review showed that trauma-focused treatment is more cost-effective than treatment as usual and no treatment. Next to the systematic review, a meta-analysis on the cost-effectiveness of interventions for adults with PTSD indicated that eye movement desensitization and reprocessing (EMDR) appeared to be the most cost-effective method, followed by trauma-focused cognitive behavioural therapy (TF-CBT) (Mavranezouli et al., 2020). Adding TF-CBT to care as usual for psychotic disorders not only led to reduced costs, but also yielded better outcomes in terms of QALY gains in patients with PTSD and a psychotic disorder (De Bont et al., 2019). Comparison of the cost-effectiveness of two adaptations of exposure therapy, i.e. i-PE and STAIR + PE, to standard PE in patients with CA-PTSD has not been done before.

In the IMPACT study, we included 149 Dutch adults with CA-PTSD. Large improvements in clinician-assessed and self-reported PTSD symptoms were observed in all three treatment conditions (Cohen’s *d* > 1.6, from baseline to 1 year follow-up), with no significant differences among conditions. i-PE led to a faster decrease in self-reported PTSD symptoms compared to PE and STAIR + PE. Also, i-PE led to a faster decrease in clinician-assessed PTSD symptoms compared to STAIR + PE, but not to PE. Moreover, dropout rates did not differ across treatment conditions. These results were obtained in a maximum of 16 sessions in 16 weeks for PE. i-PE consisted of a maximum of 12 sessions in 4 weeks and two booster sessions. STAIR + PE consisted of a maximum of eight sessions of skills training followed by a maximum of eight sessions PE in 16 weeks. Further details can be found elsewhere (Oprel et al., 2021).

### 1.1. Current study

The goal of the IMPACT study was to improve quality of care and contribute to treatment innovation for patients with CA-PTSD, while lowering societal costs (Oprel et al., 2018). The aim of the current study was to compare the cost-effectiveness of i-PE and STAIR + PE to PE in a sample of patients with CA-PTSD. Costs and effects were assessed over a 1 year follow-up period in an intention-to-treat analysis. Our a priori expectation was that i-PE and STAIR + PE would be more cost-effective, given that the treatment protocols include fewer (i-PE) and shorter (STAIR + PE) sessions.

The main results of the RCT showed that the three treatment conditions did not differ in effectiveness (Oprel et al., 2021); therefore, we nuanced our hypothesis: it is expected that i-PE and STAIR + PE are equally effective at lower cost compared to PE.

## 2. Method

### 2.1. Participants and study design

In total, 149 patients participated in an RCT that compared the effectiveness of PE, i-PE, and STAIR + PE. Detailed information on the study design and procedures of the IMPACT study can be found in the published study protocol (Oprel et al., 2018) and the published article on the main outcomes of the RCT (Oprel et al., 2021). The study sample consisted of adults with at least moderately severe PTSD related to childhood sexual and/or physical abuse (before the age of 18 years) committed by a primary caretaker or an authority figure. Participants had to (1) be between 18 and 65 years old, (2) be fluent in Dutch, and (3) have a PTSD diagnosis established with the Clinician-Administered PTSD Scale (CAPS-5) based on the Diagnostic and Statistical Manual of Mental Disorders, Fifth Edition (DSM-5) classification, and a CAPS-5 score of ≥ 26. Exclusion criteria were (1) ongoing litigation concerning their admission or stay in the Netherlands, (2) pregnancy, (3) presence of severe non-suicidal self-injury or suicidal behaviour in the past 3 months which required hospitalization, (4) presence of severe alcohol- or drug-related disorder in past 3 months, (5) cognitive impairment (IQ < 70), (6) engagement in other psychological treatment, and (7) changes in psychotropic medication during the past 2 months. Patients were recruited at two outpatient mental health services specializing in the treatment of trauma-related disorders, located in the Hague and Rotterdam, the Netherlands. Participants were informed about the study and gave their full written consent. Study procedures were approved by the Medical Ethics Committee of Leiden University Medical Center (NL57984.058.16).

### 2.2. Treatment conditions

All participants received exposure-based interventions. In the PE condition, patients received 16 treatment sessions of each 90 min (1440 min in total). Sessions included imaginal exposure involving repeated and systematic recounting of the most distressing traumatic memories and exposure in vivo involving approaching trauma-related stimuli. Patients listened to audiotapes of the imaginal exposure between sessions and practised approaching trauma-related stimuli. In the i-PE condition, participants received 14 sessions of 90 min (1260 min in total). i-PE was delivered in three sessions per week for 4 weeks followed by two booster sessions. Session content was similar to the standard PE condition, but the treatment was delivered by two alternating therapists. In the STAIR + PE condition, patients received eight sessions of 60 min skills training followed by eight PE sessions of 90 min (1200 min in total), which was delivered in 16 weekly sessions by one therapist (Cloitre et al., 2002). During the first phase (STAIR; eight sessions), emotional regulation and interpersonal skills training was provided, while the second phase of treatment included standard PE (eight sessions), similar to the PE and i-PE conditions.

### 2.3. Outcome measures

We estimated 1 year costs from the societal perspective, reported here in euros at the price level for the year 2020, when €1.00 was equated with US$1.29 (https://data.oecd.org/conversion/purchasing-power-parities-ppp.htm). Intervention costs were estimated from the participants’ actual number of sessions, with the price per session chosen to match the national tariff for treatment of anxiety disorders (Zorginstituut Nederland, 2015).

Healthcare use was measured using the Trimbos/iMTA questionnaire for Costs associated with Psychiatric Illness (TiC-P, short version) (Hakkaart-van Roijen, 2002). The TiC-P is commonly applied in the economic evaluation of treatment in mental healthcare, including trials on the cost–utility of brief psychological treatment for anxiety (e.g. de Bont et al., 2013; Hakkaart-Van Roijen et al., 2006; IJff et al., 2007). The TiC-P is a valid and reliable measure, in terms of interrater, construct, and test–retest reliability, for collecting data on healthcare consumption and productivity losses (Bouwmans et al., 2013). Participants completed the TiC-P at baseline, post-treatment (16 weeks), and at 6 and 12 months’ follow-up. Participants reported on their visits [specialists, general practitioner (GP), physiotherapist, paramedical professionals, and alternative healthcare], admissions to hospital, home care, paid domestic help, informal care, medication, and out-of-pocket expenses. Healthcare volumes (e.g. visits to the GP, sessions with a psychologist, days in a hospital) were valued using unit reference prices, designed to enhance comparability with other health-economic evaluations in the Netherlands (Kanters et al., 2017). Medication costs were obtained from the database provided by the National Health Care Institute (www.medicijnkosten.nl), assuming usage in accordance with the daily defined dosage. Medical costs included the costs for the intervention, family doctor/GP visits, mental health service visits, hospital visits, paramedical care visits, professional home care hours, informal care hours, and pharmacology. Non-medical costs included travel costs to visit health services and productivity losses in both paid and unpaid work. Travel costs were estimated assuming average travel distances for a return trip from home to the nearest health service and the prices of public transport per kilometre. Productivity costs were estimated from the number of workdays lost due to absenteeism and work cutback (presenteeism) as tallied by the TiC-P questionnaire, and productivity losses were valued using the friction-cost method, in accordance with the Dutch guidelines for economic evaluations (Kanters et al., 2017). For hourly wage, we used the national average of €38.00 per hour in 2020. Unpaid productivity was valued at €15.31 per hour.

Patients reported their health-related quality of life (HRQoL) on the 5-level EuroQol 5 Dimensions (EQ-5D-5L) (Feng et al., 2021; König et al., 2007). The EQ-5D-5L consists of the dimensions ‘mobility’, ‘self-care’, ‘usual activities’, ‘pain/discomfort’, and ‘anxiety/depression’, rated on a five-point Likert scale. Utilities were calculated from the EQ-5D-5L responses using the Dutch tariff (Versteegh et al., 2016). Utilities represent HRQoL on a scale anchored at 0 (a health state as poor as death) and 1 (perfect health). In addition, participants rated their HRQoL on the EuroQol’s visual analogue scale (VAS), which ranges from 0 (worst imaginable health) to 100 (best imaginable health). VAS scores were transformed to a utility scale using the power transformation 1 − (1 − VAS/100)_1.61_ (Stiggelbout et al., 1996)_._ EQ-5D-5L and the VAS measurements were obtained at baseline, at 4, 8, and 16 weeks after randomization, and at 6 and 12 month follow-up. Based on these utility measurements, QALYs were calculated as the area under the utility curves over the full 12 month follow-up time.

### 2.4. Analyses

Analyses were performed according to the intention-to-treat principle, using the IBM SPSS software (version 25; IBM Corp., Armonk, NY, USA). To prevent bias from possibly selective loss to follow-up, missing data were accounted for using multiple imputations with predictive mean matching. The following predictors were included in the imputation model: treatment condition, age, sex, educational level, cultural background, severity of PTSD symptoms based on the CAPS-5 score, post-traumatic symptom severity as measured with the PTSD Checklist for DSM-5 (PCL-5), depression severity as measured with the Beck Depression Inventory, second edition (BDI-II-NL) (Beck et al., 1996), and number of comorbid axis-I disorders based on the Mini-International Neuropsychiatric Interview (MINI) (Sheehan et al., 1998). Unequal-variance *t*-tests were used to assess differences in mean outcome in the i-PE and the STAIR + PE groups, compared to the PE group. The sample size calculation of the IMPACT study was based on the clinical primary outcome measure, i.e. PTSD severity, while economic outcomes were secondary. As such, inferences from the cost-effectiveness analyses are based on medical decision-making probability statements.

For the health-economic evaluation, incremental net–benefit regression analysis (INBRA) with adjustment for baseline imbalance in utility was used. Group differences in costs were related to group differences in QALYs using cost-effectiveness acceptability curves. Depending on the willingness to pay (WTP) per QALY, acceptability curves show the probability that each of the conditions is economically preferred over the other two conditions, i.e. the probability that the condition has the highest net benefit (NB = WTP × QALYs – Costs). Bootstrapping was used to estimate these acceptability curves (Hoch, Rockx & Krahn, 2006), using Stata software (version 14.2; StataCorp, College Station, TX, USA). In accordance with the Dutch guidelines for economic evaluations in healthcare, the base-case cost–utility analysis was from the societal perspective, i.e. comparing total societal costs to QALYs estimated from the EQ-5D-5L (Kanters et al., 2017). Two sensitivity analyses were performed, one restricting costs to the intervention costs only (which may be relevant for decision making at the level of mental healthcare providers) and the other using QALYs estimated from the VAS (thus taking the patients’ perspective on health).

In the Netherlands, the WTP ceiling for gaining one QALY ranges from €20,000 to €80,000, depending on the severity of the condition at hand (Zorginstituut Nederland, 2015). PTSD can be considered as a severe anxiety disorder, with a disability weight of 0.523 [95% confidence interval (CI) 0.356, –0.684] (Salomon et al., 2012). As such, the maximum WTP for gaining one QALY in PTSD would be €50,000. In other words, costs below €50,000 per QALY can be regarded as acceptable from a cost-effectiveness point of view.

## 3. Results

### 3.1. Sample characteristics

The baseline characteristics of the included participants (*N* = 149) per condition are presented in Table 1. The i-PE and standard PE conditions consisted of 14 and 16 sessions, respectively. The average number of completed treatment sessions per patient for PE, i-PE, and STAIR + PE was 11.2, 10.9, and 13.8, respectively. The STAIR sessions were shorter, i.e. 60 min, compared to the PE sessions, i.e. 90 min. Despite that, the total number of treatment minutes was, on average, not different between conditions (PE = 1011, STAIR + PE = 1038, i-PE = 981; PE vs STAIR + PE *t *= −0.35, *p = .*72, PE vs i-PE *t *= 0.33*, p = .*75).

**Table 1.** Baseline characteristics of the study sample (*N* = 149).

	Total (*N* = 149)	PE (*n* = 48)	i-PE (*n* = 51)	STAIR + PE (*n* = 50)
Demographics				
Age (years), mean (*SD*)	36.9 (11.8)	34.5 (11.1)	38.9 (11.6)	37.1 (12.4)
Female gender, *n* (%)	114 (76.5)	37 (77.1)	38 (74.5)	39 (78.0)
Education, *n* (%)^a^				
Low	56 (37.6)	16 (33.3)	21 (41.2)	19 (38.0)
Moderate	63 (42.3)	23 (47.9)	18 (35.3)	22 (44.0)
High	30 (20.1)	9 (18.8)	12 (23.5)	9 (18.0)
Job, *n* (%)				
Employed	57 (38.3)	19 (39.6)	21 (41.2)	17 (34.0)
Incapacitated/on disability	37 (24.8)	14 (29.2)	7 (13.7)	16 (32.0)
Unemployed	27 (18.1)	8 (16.7)	13 (25.5)	6 (12.0)
Student	17 (11.4)	4 (8.3)	4 (7.8)	9 (18.0)
Staying at home for children	11 (7.4)	3 (6.3)	6 (11.8)	2 (4.0)
Non-Western cultural background, *n* (%)^b^	65 (43.3)	20 (41.2)	19 (37.3)	26 (52.0)
Clinical characteristics				
CAPS-5 total score, mean (*SD*)	41.4 (9.4)	41.3 (9.8)	39.3 (7.6)	43.6 (10.5)
PCL-5 total score, mean (*SD*)	50.0 (12.7)	51.2 (12.4)	48.6 (13.1)	50.3 (15.4)
PTSD duration (years), mean (*SD*)	15.1 (12.5)	15.3 (10.2)	15.4 (12.9)	14.5 (14.2)
Any personality disorder, *n* (%)	90 (60.4)	33 (68.8)	26 (51.0)	31 (62.0)
MINI diagnoses, mean no. excl. PTSD (*SD*)	3.1 (1.9)	3.2 (1.9)	2.8 (1.8)	3.4 (2.0)
Psychotropic medication, *n* (%)	71 (47.7)	24 (50.0)	25 (49.0)	22 (44.0)
Economic characteristics				
Utility, EQ-5D-5L, mean (*SD*)	0.489 (0.279)	0.437 (0.340)	0.498 (0.243)	0.530 (0.247)
Utility VAS, mean (*SD*)	56.4 (24.3)	60.3 (24.9)	58.57 (21.7)	60.3 (24.9)

Note: PE, prolonged exposure; i-PE, intensified prolonged exposure; STAIR + PE, Skills Training in Affective and Interpersonal Regulation + prolonged exposure; CAPS, Clinician-Administered PTSD Scale; PCL-5, PTSD Checklist for DSM-5; PTSD, post-traumatic stress disorder; MINI, Mini-International Neuropsychiatric Interview; EQ-5D-5L, 5-level EuroQol 5 Dimensions; VAS, visual analogue scale.

^a^ Low: primary education or lower general secondary education; moderate: intermediate vocational education; high: higher vocational education or university.

^b^ Non-Western cultural background: at least one parent was not born in a Western country.

### 3.2. Dropout

Demographic and clinical characteristics were not related to dropout from therapy. Change in PTSD symptoms from baseline to week 4 did not predict subsequent therapy dropout. Little’s MCAR test indicated that missing cases met the criteria for being missing completely at random (*χ*^2^(244) = 241, *p* = .54). Regarding data on the main outcomes of the present study (TiC-P, EQ-5D-5L, and VAS), 14.2% was missing at T1, 22.8% was missing at T2, 35.6% was missing at T3 and T4, and 37.6% was missing at T5.

### 3.3. Main analyses

All the results of the main analyses can be found in Table 2.

**Table 2.** Average volumes, costs, and quality-adjusted life-years (QALYs) per patient, by condition.

	Average volume (*SE*)			Average cost (€) (*SE*)			PE vs i-PE		PE vs STAIR + PE	
	PE	i-PE	STAIR + PE	PE	i-PE	STAIR + PE	*t*	*p*	*t*	*p*
Medical costs										
Intervention visits	11.2 (0.71)	10.9 (0.71)	13.8 (0.60)	4479 (196)	4389 (197)	4464 (164)	0.33	.745	0.06	.952
Family doctor/GP visits	4.2 (0.88)	5.7 (1.6)	4.5 (0.88)	151 (32)	204 (59)	160 (32)	−0.80	.426	−0.21	.821
Mental health service visits	2.6 (0.96)	5.9 (1.5)	3.7 (1.2)	5634 (416)	6160 (489)	5346 (300)	−0.82	.414	0.56	.574
Hospital visits	5.1 (1.0)	3.5 (0.7)	4.7 (1.0)	892 (285)	281 (75)	391 (109)	2.07	.038*	1.64	.101
Paramedical care visits	4.9 (1.8)	3.0 (1.1)	2.9 (0.85)	211 (68)	207 (84)	172 (50)	0.04	.971	0.46	.645
Professional home care (hours)	16.8 (10.2)	0.7 (0.7)	30.1 (26.0)	683 (425)	18 (18)	769 (652)	1.56	.118	−0.11	.912
Informal care (hours/week)	2.2 (0.66)	2.3 (1.1)	1.9 (0.68)	33 (10)	35 (16)	29 (10)	−0.10	.918	0.26	.797
Pharmacology				466 (90)	487 (122)	447 (85)	−0.14	.893	0.16	.873
Total medical costs				7888 (888)	7152 (539)	7125 (924)	0.71	.479	0.60	.552
Non-medical costs										
Work loss (absenteeism) (hours)	157.2 (60.2)	180.4 (70.2)	165.0 (49.1)							
Work cutback (presenteeism) (hours)	46.6 (14.1)	75.3 (16.8)	73.2 (16.5)							
Paid work loss costs				7742 (2594)	9714 (3031)	9053 (2247)	−0.49	.621	0.38	.702
Unpaid work loss (days)	7.7 (2.1)	9.6 (2.2)	7.4 (2.1)	2144 (738)	2544 (629)	1959 (637)	−0.41	.680	0.19	.849
Travel costs				108 (18)	136 (25)	90 (16)	−0.96	.339	0.76	.447
Total non-medical costs				10027 (2756)	12429 (3409)	11131 (2483)	−0.55	.584	−0.30	.766
Total societal costs	** **			17914 (3114)	19581 (3735)	18256 (2842)	−0.34	.732	−0.08	.935
QALYs										
QALYs based on EQ-5D-5L	0.778 (0.018)	0.805 (0.018)	0.794 (0.016)				−1.07	.284	−0.63	.530
QALYs based on VAS	0.767 (0.023)	0.847 (0.013)	0.800 (0.016)				−3.03	.002*	−1.16	.245

Note: PE, prolonged exposure; i-PE, intensified prolonged exposure; STAIR + PE, Skills Training in Affective and Interpersonal Regulation + prolonged exposure; GP, general practitioner; QALY, quality-adjusted life-year; EQ-5D-5L, 5-level EuroQol 5 Dimensions; VAS, visual analogue scale.

*Significant difference between groups (*p* < .05).

#### 3.3.1. Medical costs

Intervention costs did not differ between PE versus i-PE (€90, 95% CI −454, 635) and PE versus STAIR + PE (€15, 95% CI −486, 516). Compared with PE, costs for hospital visits were significantly lower in the i-PE condition (€611, 95% CI 36, 1186). All other types of medical costs and total medical costs did not differ between PE versus i-PE (€−736, 95% CI −1300, 2770) and PE versus STAIR + PE (€−763, 95% CI −1722, 3247).

#### 3.3.2. Societal costs

Paid and unpaid productivity loss and travel costs in the i-PE and STAIR + PE condition did not differ from those in the PE condition. Also, total societal costs did not differ between PE versus i-PE (€−1667, 95% CI −1300, 2770) and PE versus STAIR + PE (€342, 95% CI −1722, 3247).

#### 3.3.3. Utilities and QALYs

**Figure 1.**
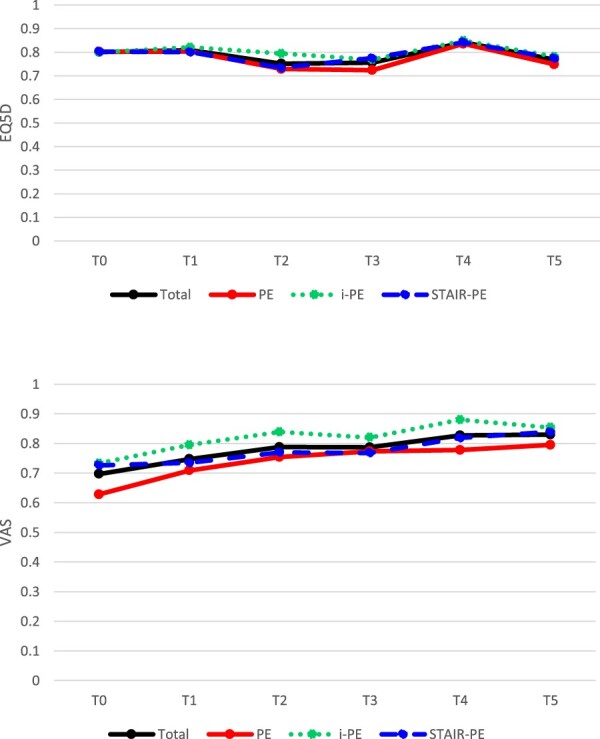
Average utility through the year, according to (A) the 5-level EuroQol 5 Dimensions (EQ-5D-5L) and (B) the visual analogue scale (VAS). T0 = Baseline; T1 = after 4 weeks (4 sessions of STAIR + PE or PE or 12 sessions of i-PE); T2 = after 8 weeks (8 sessions of STAIR + PE or PE or 13 sessions of i-PE); T3 = after 16 weeks (i.e. post-treatment); T4 = 6 month follow-up (26 weeks); T5 = 12 month follow-up (52 weeks). PE, prolonged exposure; i-PE, intensified prolonged exposure; STAIR, Skills Training in Affective and Interpersonal Regulation.

Utilities showed similar patterns across the three interventions throughout the 1 year follow-up period (Figure 1). QALYs based on the EQ-5D-5L were similar in the three conditions (Table 2). QALYs according to the VAS were higher in the i-PE (*t *= −3.03, *p *= .002) compared with the PE condition. As a sensitivity analysis, we tested whether VAS-based QALYs in the i-PE condition differed from the PE condition while controlling for patient-reported utilities prior to treatment. Hence, we added baseline VAS scores to the regression model as a covariate. The results showed that VAS-based QALYs remained higher in the i-PE compared to the PE condition (*t *= 2.36, *p *= .019).

#### 3.3.4. Cost–utility analysis

**Figure 2.**
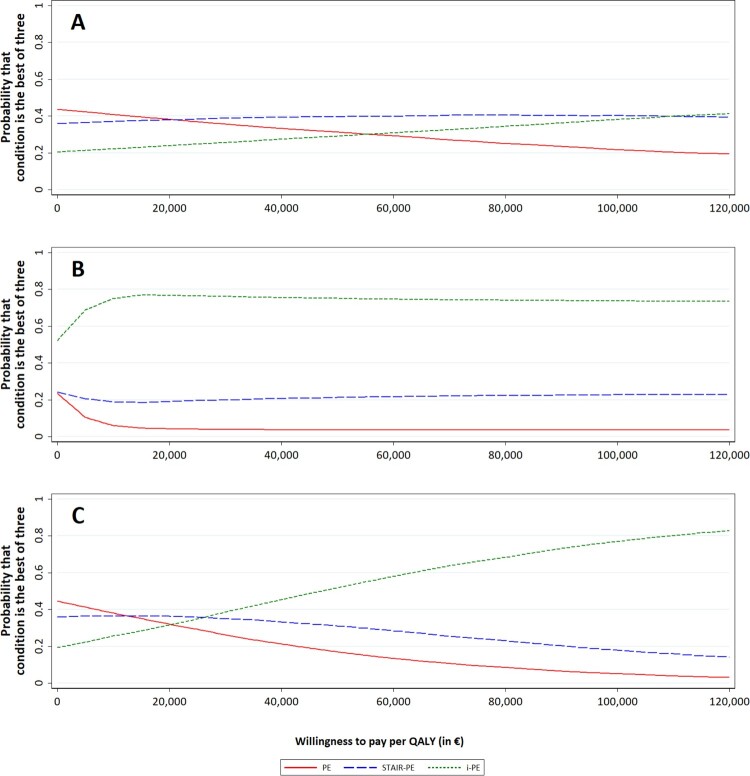
Cost-effectiveness acceptability curves, i.e. the probability that each of the conditions is economically preferred over the other two conditions, depending on the willingness to pay per quality-adjusted life-year (QALY). (A) Base-case analysis, based on societal costs and QALYs estimated from the 5-level EuroQol 5 Dimensions (EQ-5D-5L); (B) sensitivity analysis based on intervention costs (instead of societal costs) and QALYs estimated from the EQ-5D-5L; (C) sensitivity analysis based on societal costs and QALYs estimated from the visual analogue scale (instead of the EQ-5D-5L).

In the primary economic evaluation, societal costs were related to the QALYs based on the EQ-5D-5L. Acceptability curves show the probability that each of the three treatments (PE, i-PE, STAIR + PE) has the best efficiency, depending on the WTP for a QALY. As shown in Figure 2(A), the primary acceptability curve indicated that differences between the three treatment conditions were negligible. Only some small differences could be detected. That is, based on the lower societal costs, PE was slightly more advantageous at a low WTP. Patients in the i-PE condition had slightly more QALYs based on the EQ-5D-5L, and i-PE was therefore more favourable at a high WTP. Moreover, the STAIR + PE was preferred in the mid-range of WTP. However, at the WTP threshold of €50,000 per QALY, STAIR + PE has a 40% likelihood of being the optimal condition, which is not much higher than the values of 32% and 28% found for the PE condition and the i-PE condition, respectively. Based on Figure 2(A), it can be concluded that, across the total range of WTP, the probability of being optimal does not differ much between treatment conditions.

Figure 2(B) shows the sensitivity analysis in which the costs are restricted to only the intervention costs (thus assuming no difference in other costs). As the difference in intervention costs is small, this analysis mostly reflects the difference in QALYs, which is in favour of the i-PE condition. i-PE is the only one of the three interventions that has a > 50% likelihood of being optimal at WTP = 0. Also, at €50,000 per QALY, i-PE has a 74% likelihood of being the optimal condition. Figure 2(C) shows the sensitivity analysis in which the QALYs are estimated using the VAS, which is more in favour of the i-PE condition compared to PE. At €50,000 per QALY, i-PE has a 52% likelihood of being the optimal condition.

## 4. Discussion

The results of the present cost-effectiveness analyses indicate that the three forms of PE do not differ in costs, when taking the societal perspective. In addition, differences in gains in QALYs based on the EQ-5D-5L between treatments were negligible. As such, in light of medical decision making, the overall impression is one of equivalence of the three interventions. Only some small differences between treatment conditions could be observed. In the PE condition, societal costs were lower compared to the other conditions and this is therefore the preferred treatment option at a low WTP. At a high WTP, however, gains in QALYs based on the EQ-5D-5L were slightly in favour of the i-PE treatment condition. Also, (non-mental) healthcare costs and QALYs based on the VAS were in favour of i-PE, possibly owing to the shorter period of active treatment duration. Between-person differences could play a role in the group differences, such as the considerable individual differences in professional home care hours and work loss, which are also reflected by the relatively large standard errors. Despite these findings in favour of (i-)PE, a pronounced preference for any one of the three evidence-based treatments cannot be made based on the main outcomes of our economic evaluation, i.e. total societal costs and QALYs based on the EQ-5D-5L.

Based on the findings of the current health-economic evaluation, i-PE and STAIR + PE could therefore be treated as equivalent to PE by policy makers when considering implementation. It is well known that treatment preference positively influences patients’ satisfaction as well as treatment outcomes and dropout (Windle et al., 2020). Given the lack of differences in clinical outcomes (Oprel et al., 2021) and cost-effectiveness among treatments, as found in the present study, our findings leave room for shared decision making in choosing PTSD treatment, taking into account the preferences of patients and clinicians (Hamblen et al., 2019). Despite the lack of differences in clinical outcomes, it was previously found in the current study sample that several clinical predictors could indicate a more optimal treatment choice (Hoeboer et al., 2021). As such, personalization offers a promising method by which to assign patients to their optimal treatment (Deisenhofer et al., 2018; Keefe et al., 2018).

The cost-effectiveness of i-PE and STAIR + PE compared to PE has not been studied until now. Although we were unable to compare the three exposure therapies to treatment as usual and no treatment, it could be assumed that all three variants of exposure therapy are more cost-effective than treatment as usual and no treatment, based on previous findings (von der Warth et al., 2020).

### 4.1. Strengths, limitations, and future studies

This is the first study to evaluate the cost-effectiveness of three PE-based interventions for patients with CA-PTSD. The pragmatic and naturalistic design of the study enhances the generalizability of the results to clinical practice. Moreover, missing values on outcome variables were estimated using multiple imputation to retain the full sample size and use as much information from the data as possible.

Next to these strengths, limitations need to be acknowledged. First, patients’ healthcare usage and productivity losses were based on self-reports. Self-report registrations may be more sensitive to errors than data from healthcare services and employers. Yet, it was previously shown that agreement between patient’s self-reported healthcare usage and data provided by health services was high (Bouwmans et al., 2013). Secondly, statistical power calculations to detect differences between the three treatment conditions were based on the clinical outcomes and not on the costs and QALYs. As such, the lack of significance of our findings from between-group comparisons may be due to underpowering. Although HRQoL improved slightly from baseline to post-treatment in all conditions (Walters & Brazier, 2005), the EQ-5D-5L measure in our base-case analysis did not seem to pick up the improvement over time that we found for the clinical outcomes (Oprel et al., 2021). This could be due to the fact that the dimensions assessed with the EQ-5D reflect general (or physical) health-related quality of life rather than mental well-being. In this context, the acceptability curves are appropriate to make probabilistic statements to draw health-economic conclusions. Thirdly, generalizability of the results may be limited owing to differences in healthcare systems across settings and countries. We recommend replication in different mental healthcare settings and in other populations, e.g. PTSD related to traumatic events other than childhood abuse. Moreover, comparing cost-effectiveness of the exposure-based interventions, as applied in the present study, to other guideline-recommended treatments for PTSD, such as EMDR and imagery rescripting, for patients with CA-PTSD in future studies may elucidate which of these recommended treatments is most cost-effective in the CA-PTSD group. Also, future studies could investigate barriers to the implementation of diverse trauma-focused treatments, such as reimbursement and the organization of training and supervision of therapists in different settings.

## 5. Conclusion

Exposure-based treatment in patients with CA-PTSD yields considerable personal and societal economic benefits in this relatively costly and severely affected patient group. The current findings showed that the three exposure-based treatments do not differ in terms of outcomes and costs. As described elsewhere, the findings on the main clinical outcomes from the RCT showed that all treatment conditions resulted in large improvements in PTSD symptoms from baseline to 1 year follow-up (Cohen’s *d* > 1.6) and that there were no significant differences between treatments (Oprel et al., 2021). The absence of marked differences in costs and clinical outcomes among treatment conditions indicates that shared decision making can be based on other factors, to meet individual patient preference regarding treatment choice.

## Data Availability

Proposals for the use of data and requests for access should be directed to vanderdoes@fsw.leidenuniv.nl.
